# Hemispheric Asymmetries in Electroencephalogram Oscillations for Long-Term Memory Retrieval in Healthy Individuals

**DOI:** 10.3390/brainsci10120937

**Published:** 2020-12-04

**Authors:** Soyiba Jawed, Hafeez Ullah Amin, Aamir Saeed Malik, Ibrahima Faye

**Affiliations:** 1 Centre of Intelligent Signal and Imaging Research & Department of Electrical and Electronic Engineering, Universiti Teknologi PETRONAS, Seri Iskandar 32610, Malaysia; soyiba.jawed_g03701@utp.edu.my; 2 School of Computer Science, Faculty of Science and Engineering, University of Nottingham Malaysia, Semenyih 43500, Malaysia; hafeezullah.amin@nottingham.edu.my; 3 Department of Electrical and Electronics Enginnering, University of Jeddah, Jeddah 23218, Saudi Arabia; aamirdip@gmail.com; 4 Centre of Intelligent Signal and Imaging Research & Department of Fundamental and Applied Sciences, Universiti Teknologi PETRONAS, Seri Iskandar 32610, Malaysia

**Keywords:** hemispherical encoding retrieval asymmetry, electroencephalography, long-term memory, recent long-term memory, remote long-term memory

## Abstract

The hemispherical encoding retrieval asymmetry (HERA) model, established in 1991, suggests that the involvement of the right prefrontal cortex (PFC) in the encoding process is less than that of the left PFC. The HERA model was previously validated for episodic memory in subjects with brain traumas or injuries. In this study, a revised HERA model is used to investigate long-term memory retrieval from newly learned video-based content for healthy individuals using electroencephalography. The model was tested for long-term memory retrieval in two retrieval sessions: (1) recent long-term memory (recorded 30 min after learning) and (2) remote long-term memory (recorded two months after learning). The results show that long-term memory retrieval in healthy individuals for the frontal region (theta and delta band) satisfies the revised HERA asymmetry model.

## 1. Introduction

In neuroscience, the term “memory” is defined as a combination of three components, namely, encoding, storage, and retrieval. Studies have been conducted to develop techniques targeting neural substrates with varying specificities. Brain asymmetry is a well-discussed topic in neuroscience. Many studies have proved that high functional processes, including memory and learning, are dependent on the hemisphere of the brain [1].

Since the early 1990s, several research works have been carried out based on memory tests using brain signal capturing techniques such as positron emission tomography (PET) and functional magnetic resonance imaging (fMRI) to confirm the engagement of different brain regions in cognitive tasks. This behavior was observed by researchers [2,3] in subjects performing learning tasks. The results showed greater involvement of the left prefrontal cortex (PFC) than the right PFC in performing learning tasks. However, the opposite has been observed true in the case of memory retrieval. During memory retrieval, the left PFC shows lower involvement than the right PFC [3]. Memory encoding is fundamental to the hemispheric encoding retrieval (HERA) model [2,3]. Studies based on fMRI, event-related potential (ERP), and PET have shown that the frontal neuronal network [4] in adults and young individuals is significantly involved in long-term semantic memory. The right and left prefrontal areas of the brain represent long-term memory perspectives, such as episodic and semantic long-term memory, that manage different sets of information [5]. Episodic long-term memory represents autobiographical events, whereas semantic long-term memory represents knowledge and learning contents [6]. The brain signal capturing technique, electroencephalography (EEG), was used in some studies to investigate long-term memory synchronization and desynchronization [6,7]. In particular, (1) for the upper alpha (9.5–13 Hz) band, desynchronization is associated with semantic memory, and (2) the retrieval and search processes are indicated in the semantic memory by the upper alpha oscillations [6]. The encoding of new information for thalamocortical feedback loops is shown by theta (4–7 Hz) oscillations in the hippocampus-cortical [6]. Long-term memory can be divided into two categories [7]: (1) explicit long-term memory [7] and (2) implicit long-term memory [8]. In previous studies, category learning [8,9], a type of implicit long-term memory, was explored. In another review [10], neuroimaging revealed activation with a specific area of the prefrontal cortex in subjects with brain injuries. The analysis found the number of identities that can be missed if the prefrontal cortex is treated as a single entity, which is the basis of the HERA model. The study highlighted that the prefrontal cortex should not be considered as a single entity, because the roles of individual areas in the prefrontal cortex can be overlooked.

A revised formulation of HERA model [6] considered the encoding and retrieval phases independently or together. In this study, the right asymmetry in the gamma and theta bands using the HERA model was observed. Although the HERA model is not an absolute feature for long-term memory models, it is affected by the type of learning material. As reported in the previous studies [6], the nature of learning materials such as video stimuli plays a significant role in PFC activation in comparison tasks (verbal versus non-verbal) rather than memory processes (encoding versus retrieval) [6]. Therefore, learning materials are of significant importance and the results are affected by the nature of the learning material, including difficulty level, content, and details. Absolute measures of brain activity in encoding and retrieval in each hemisphere and differences across hemispheres are irrelevant to the HERA model. Instead, it is the hemispheric asymmetries in the differences in activities between encoding and retrieval that are critical.

The main advantages of using the revised HERA model are as follows. (1) The classical HERA model requires a comparison between the retrieval and encoding phases. In the revised HERA model, the retrieval and encoding processes do not need to be tested simultaneously; a research study can be oriented on the retrieval process, encoding process, or considerably on both processes. (2) The revised formulation of the HERA model does not depend on the test materials or nature of the research study, experimental variables, and type of retrieval/encoding task.

From the above discussion, it can be seen that the focus of most existing studies has been episodic memory, which has been tested for individuals with brain traumas and injuries. In this study, the revised HERA model was tested for semantic long-term memory retrieval in healthy individuals using video stimuli under the hypothesis that the brain patterns recorded after learning new content satisfied the hemispherical functional asymmetry for semantic long-term memory retrieval in young healthy individuals, consistent with the revised HERA model. To the best of our knowledge, the HERA model has not been tested previously on young healthy individuals to analyze hemispheric asymmetries in semantic long-term memory retrieval.

In this study, the alpha (7–13 Hz), beta (13–39 Hz), theta (4–7 Hz), delta (<4 Hz), and gamma (>40 Hz) EEG bands were investigated to determine brain asymmetry using the HERA model in a long-term memory retrieval task. This model was used to evaluate the long-term memory quality for retrieval in healthy subjects who are shown learning contents in a video format. The video content was new for the subjects. A pretest was conducted before showing the video content to the subjects, to ensure that they had no information about the video content. This EEG-based technique evaluates the brain rhythms of the occipital, parietal, and frontal areas during the recent long-term memory retrieval (RLTM) and remote long-term memory retrieval (ReLTM) phases using video-based visual content. The RLTM was recorded thirty minutes after showing the videos, and ReLTM was recorded two months after. Here, the memory test was conducted to estimate the recent and remote long-term memory retrieval. The reason behind examining a video-based visual modality is its stability; it provides an exciting and enjoyable learning process for the subjects [11] compared to other visual modalities such as picture-based visual modalities. A video-based modality is significantly stable because it considers all perspectives of visual modalities. In addition, the subjects learn more if the learning content is interactive, such as in video formats, because it contains animations [12]. 

## 2. Materials and Methods

Thirty-four university students were recruited as subjects for this study (age: 18–30 years, 23.17 ± 3.04). They had no neurological disorders. Their vision was corrected to normal. Although they were right-handed, their brain regions related to handedness were not examined. The subjects were asked if they were right- or left-handed during the recruitment process. None of the subjects had hearing damage, and they signed a consent form before the experiment. The human research ethics committee of Hospital Universiti Sains Malaysia approved the protocol of this study (ref no. USMKK/PPP/JEPeM [2 57.3.(3)]) [11,13].

The experiment duration was 10 min. The EEG was recorded during the experimental tasks. The frontal, parietal, and occipital regions of the brain have been proven to be active during learning [14,15,16]. In this study, all electrodes covering these three regions were considered.

### 2.1. Sample Size Calculation

To compute the sample size, the following parameters are defined.

**Experiment:** 2-D content to observe memory recall based on learning.

**Design:** To access memory recall using 2D content, a repetitive measure design was used [13].

**Analysis:** ANOVA was used for the analysis with alpha, which is the significance level threshold, at 0.05 (one-sided). In statistics, this threshold is used to reject the null hypothesis if the null hypothesis is true. The sample size was calculated as *n* = 34 using the parameters as defined in [13]. 

### 2.2. Stimuli

The general cognitive ability varies among individuals, and it may influence the learning results between the experimental groups in the present study. Therefore, the Raven’s Advanced Progressive Matrix (RAPM) [17] test was included in this study to record the general cognitive ability of all the participants, to avoid biased results. RAPM is a standard psychometric test employed from Pearson Education, USA. It directly measures cognitive ability using two fluid components; it is a non-verbal test [17] defined as (i) “the capability to understand confusion” and (ii) “the capability to reproduce and recall information that is comprehensively explained and transferred from one to another” [17].

**Figure 1:**
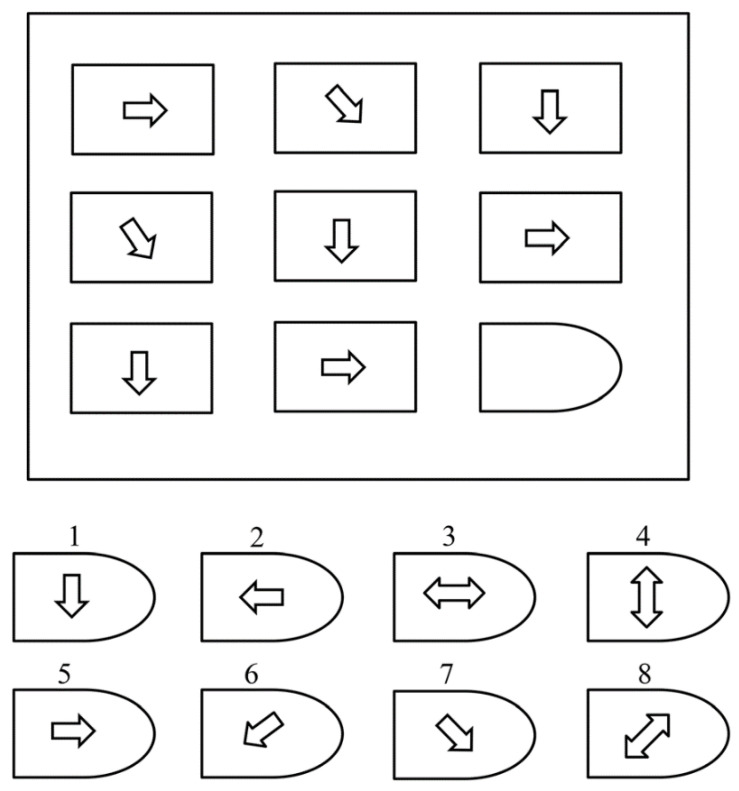
An example of a Raven’s Advanced Progressive Matrix (RAPM) problem.

The test consists of a sequence of patterns, typically 48 patterns, that are separated into two tests. We denote them as Test 1 and Test 2. Test 1 was used as a practice test, and Test 2 was used to evaluate the cognitive ability. Test 1 uses 12 patterns. Test 2 uses the remaining 36 patterns to measure the cognitive ability of the subjects. Each of the 36 patterns consists of a 3 × 3 cell structure with a depiction of the geometrical shape. However, the cell at the bottom right is excluded, leaving it void or blank; the subject can choose a correct answer from eight different options and fill in the blank. For every correct option, the subject scores “1”, and for every incorrect option, the subject scores “0”. The subjects were required to complete Test 1 in 10 min and Test 2 in 40 min [17]. Figure 1 shows an example of the RAPM test.

### 2.3. Procedure

The procedure included two main tasks: (i) a learning task and (ii) a memory retrieval task. First, the learning task was performed. This learning task included 8–10 min animated learning materials on human anatomy. The learning material was presented in video formats. The subjects had no information about the learning material. This learning material was selected to provide new knowledge to the subjects to appropriately evaluate their learning and memory skills. For the memory retrieval task, the subjects were to answer 20 questions based on the learned animated material through a pattern of multiple choices, i.e., multiple-choice questions (MCQs). Each question has four possible options, where one is the correct answer. The subjects were given 30 s to record their answers to each question.

To ensure that the subjects did not have any prior information regarding the learning material, they were required to take a pretest of 10 questions based on the learning material. The subjects that could correctly answer 10% of the pretest questions were not included in the study. All subjects were instructed on the procedure. However, no participant showed prior knowledge about the learning contents, and thus, no participant was excluded based on prior knowledge. The learning session ended with a 30 min break. The subjects were thereafter tested for retention, followed by a memory retrieval test. This was used to assess their learning performance. A 42 inch TV screen was used to display the learning task at a distance of 1.5 m from the subject, and an E-Prime Professional 2.0 tool (branded as “Psychology Software Tools, Inc., Sharpsburg, PA, USA”) [18] was used to design the task.

**Figure 2:**
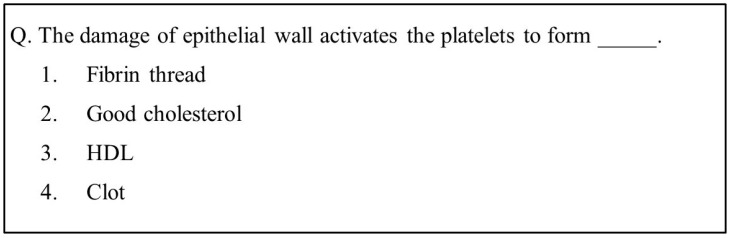
Example of a multiple-choice question.

The memory retrieval of subjects was tested using the retrieval test. In the memory retrieval task, 20 multiple-choice questions (MCQs) based on the learned animated content were presented as shown in Figure 2. Each MCQ has four possible answers, with one correct answer. The time to answer each MCQ was 30 s within a maximum time limit of 10 min. Subjects were asked to press a numeric key on the keyboard, serially numbered # 1 to # 4, corresponding to each possible answer. The maximum allowed time for this is 10 min. The first retrieval was conducted 30 min after learning session, and the second retrieval session was conducted after 2 months of the first retrieval session.

### 2.4. EEG Recording

The 128-channel HydroCel Geodesic Sensor Net (Electrical Geodesic Inc., Eugene, OR, USA) was used for continuous EEG recording. The reference electrode was Cz, which recorded the raw EEG signals that were amplified using an EGI Net Amps 300 amplifier. The impedance was under 50 kΩ, and the sampling rate was 250 Hz.

The EEG signals were filtered using an IIR filter, and the cutoff frequency was set to 0.5 Hz and 48 Hz with a roll of 12 dB per octave, which are standard values for the filter. Artifacts were removed using brain electric source analysis (BESA) software surrogate model approach. The total duration of the raw EEG recording was 10 min. The clean EEG that represents the clean data is 2–5 min long. The list of electrodes is shown in Table 1.

## 3. Results

### 3.1. Computing PSD for the HERA Model

The brain waves of the subjects were analyzed to determine the brain asymmetry (dominant brain region) of the subjects after performing two tasks: (1) learning and (2) retrieval, based on the animated visual learning material using the HERA model. A brain dominance analysis was performed for the newly learned task (RLTM) and the retrieval task (ReLTM).

The subjects were asked to answer the test questions rapidly because speed and accuracy are parameters considered for the analysis. An increasing number of incorrect answers indicates that the subject’s left brain is the dominant one. Because visual learners exhibit right-brain dominance [19], a person who is a non-visual learner will exhibit left-brain dominance. This claim is then verified using the HERA model [20].

We considered all EEG bands: alpha, beta, gamma, theta, and delta. The EEG time series [21] power spectrum was computed using fast Fourier transforms (FFT) (Welch method) with 2-s segments (2 × 250 = 500 points) and a hamming window, considering 50% overlap (i.e., certainly 250 points) and maintaining the non-equispaced FFT (NFFT) as 512 points.

The power of all the EEG bands was first computed using power spectrum density (PSD). Then, the extracted power of all the bands was input into the equation of the HERA model. The power asymmetry was tested for two retrieval sessions. The first retrieval session was recorded after 30 min of learning. The second retrieval session was recorded two months after learning. To investigate if the region (e.g., frontal, parietal, and occipital) is right-dominant or left-dominant, all the electrodes of that region were considered.

### 3.2. Evaluating the HERA Model

We evaluated the HERA model using EEG data, where the content in a video format is used to investigate the asymmetry of healthy individuals. The revised HERA model, proposed in a previous study [6] is used in this study. The revised HERA model claimed that it can investigate asymmetry independent of the learning material and type of learning tasks. In addition, the encoding and decoding conditions can be tested separately or together. In this study, a direct comparison of the two processes of HERA retrieval (1) RLTM and (2) ReLTM was made.

The right cortex is denoted as R, and the left cortex is denoted as L. The sequence of tasks can be either ReLTM or RLTM, and the R and L characterize the level of indirectly computed neuronal activity at a specified cortex for the given task.

The encoding component for the HERA model is validated if (Encoding Left – Retrieval Left) > (Encoding Right – Retrieval Right) and vice versa if (Encoding Left – Retrieval Left) < (Encoding Right – Retrieval Right). Similarly, the retrieval component for HERA is validated if (Retrieval Right – Encoding Right) > (Retrieval Left – Encoding Left), and vice versa if (Retrieval Right – Encoding Right) < (Retrieval Left – Encoding Left) [6].

In this study, the retrieval component was used for the analysis. The Hera model is validated if

(1) (ReLTM R – RLTM R) − (ReLTM L – RLTM L) > 0     

and is considered to be violated if

(2) (ReLTM R – RLTM R) − (ReLTM L– RLTM L) < 0

The inequalities show the retrieval and encoding components of the HERA model. If the condition is satisfied, the asymmetry index has a positive value, and if the condition is violated, the asymmetry index has a negative value. The extracted power of all the bands was used as input to the HERA model equation. The HERA model was applied to 31 subjects, because three subjects did not appear for retrieval session 2. The RLTM and ReLTM components of the HERA model were analyzed to observe brain dominance in the parietal, occipital, and frontal regions.

The EEG signals were analyzed for the frontal region recorded on 22 scalp sides using 11 electrode pairs. For the occipital region, EEG was recorded from two scalp sides using one electrode pair, and for the parietal region, six electrode pairs were considered. Details of the electrodes are presented in Table 1. The revised HERA model was applied to identify the dominance. For each subject, the electrode pairs were analyzed for all EEG frequency bands. The decision was formulated by combining the effect of all the electrodes for the given region.

If the revised HERA model condition is violated, the right-hand side holds a higher value. If it is satisfied, the left-hand side holds a higher value. This can be represented by the positive or negative value of the difference as shown in Equations (1) and (2). Table 2 illustrates the mean ± SD values for the HERA model equation for the parietal, frontal, and occipital regions for all subjects.

### 3.3. Statistical Significance of the HERA Model

Statistical comparisons were performed by applying a one-sample t-test to evaluate the HERA model. The PSD coefficients of three scalp regions for five bands were computed for each subject. The test results showed that the null hypothesis of the data of the bands (alpha, beta, gamma, theta, and delta) has unknown variance and zero mean. The alternative hypothesis is that the mean is positive. The PSD values for each EEG band (alpha, beta, gamma, theta, and delta) were tested for the retrieval condition. The regions of interest are the parietal right (PR), parietal left (PL), frontal right (FR), frontal left (FL), occipital left (OL), and occipital right (OR). We focused on investigating the effects of brain waves on the hemispheric functional asymmetry strictly predicted by the HERA model. In the frontal region, *t*(31) = 1.03, *p* = 0.307 for the alpha band (M = −4.97, SD = 26.71), *t*(31) = 0.86 and *p* = 0.392 for the beta band (M = −20.21, SD = 129.68), and *t*(31) = 1.06, *p* = 0.295 for the gamma band (M = −36.61, SD = 194.72), indicating no evidence of coordination with the HERA model. In contrast, in the frontal region (using FR and FL), for the theta band (M = 13.44, SD = 24.99), *t*(31) = 3.04, *p* = 0.004, and for the delta band (M = 84.52, SD = 35.95), *t*(31) = 13.30, *p* = 0.0001; the differences were statistically significant (Table 3). These results show that when using PSD, the HERA model for the theta and delta bands of the frontal region was satisfied. The results for the other regions are presented in Table 4 and Table 5.

### 3.4. HERA Model with a Topographical View

**Figure 3:**
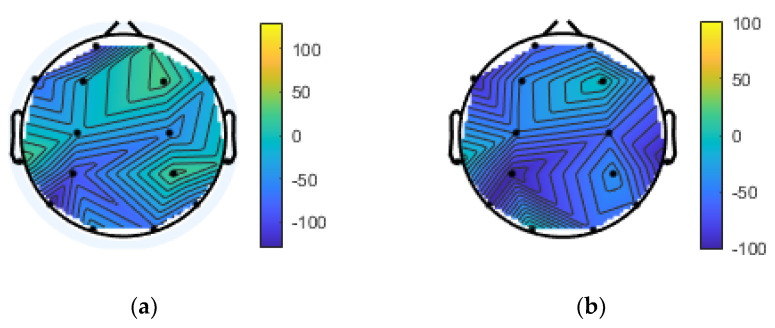
Topographical plot of theta frontal region. (**a**): left side of the frontal region representation for the HERA retrieval equation; (**b**): right side of the frontal region representation for the HERA retrieval equation.

**Figure 4:**
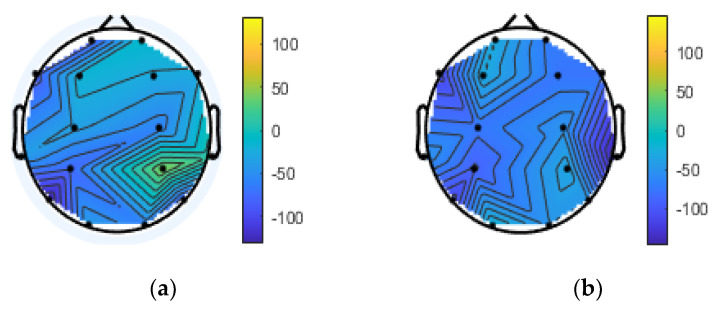
Topographical plot of delta frontal region. (**a**): left side of frontal region representation for the HERA retrieval equation; (**b**): right side of the frontal region representation for the HERA retrieval equation.

The topographical plot of the theta and delta bands of the frontal electrode satisfies the condition of the HERA model for PSD asymmetry features (shown in Figure 3 and Figure 4). Here, the encoding condition and the retrieval condition are characterized by the theta and delta bands, respectively. These results are not consistent with the results of previous studies [19], indicating that the theta and alpha bands are activated in the long-term memory retrieval process.

## 4. Discussion

In this study, we validated brain rhythmicity using the HERA model based on learned tasks. The HERA model was satisfied using the retrieval condition for the theta and delta bands of the frontal region. Most previous studies associate the changes in alpha and beta band with learning. However, some existing studies hypothesized that the delta band also shows interesting changes associated with learning [22,23]. Additionally, many previous studies have shown that theta and delta bands of the frontal region are active during learning, particularly during decision-making processes [24]. The theta and delta bands play an important role in information exchange during visual learning and cognitive tasks [25,26]. Thus, the activation of the theta and delta rhythm during learning is supported by the results of previous studies [25,26].

The authors who developed the HERA model claimed that it can be satisfied with different types of information presented to subjects in any visual modality. Our study is unique in the sense that it explored s video-based visual modality consisting of animation content to determine the asymmetry of the brain using the HERA model for long-term memory retrieval in healthy individuals. It differs from previous EEG-based studies wherein visual modalities, such as pictures, were used to determine the asymmetry of the brain using the HERA model [20]. We used two long-term memory retrieval phases, using two-time epochs. The first (RLTM) was 30 min after learning, whereas the second (ReLTM) was two months after learning.

In this study, activation was observed in the frontal region for the theta and delta bands, in healthy subjects who were shown video-based learning material for a long duration of retrieval.

The limitation of the above observations is that it is not suitable for different topographies of responses in retrieval conditions, even when video features are used. This observation explains that the presented frontal theta and delta responses can describe the sensory-perceptual processing side of the retrieval processes. In the first retrieval phase, the left frontal theta and delta indicate the representation of a visual event. In the second retrieval phase, the right frontal theta and delta responses match the actual stimulus with the sensory representations formed by the first retrieval processes. This stage of processing represents a similar HERA model, such as a sensory model for neuronal synchronization. This can explain the prominent responses of bands such as theta and delta in the frontal region. The parietal and occipital synchronization did not satisfy the HERA model because the approach did not fully engage in brain activities in these regions. This means significant brain-expressing modalities are required to show the HERA model in this study. Using a combination of different neurophysiological techniques for exploring brain activity in successful long-term memory retrieval can contribute to addressing the parietal and occipital processes on the basis of the outer layer of the brain functional asymmetries. The idea of a unique pattern of the outer layer activation of the brain explaining the long-term memory retrieval processes appears limited and dependent on the techniques used to investigate the brain functions. Alternatively, a comprehensive cognitive model that considers several attributes of parallel functional processing, including hemodynamics, blood flow, and brain rhythmicity, will be useful when investigating the exact multidimensional techniques of the working brain.

## 5. Conclusions

This EEG brain-capturing modality-based study on healthy university students showed that the HERA model is satisfied for the frontal theta and delta bands in a long-term memory retrieval task. The goal was to investigate if brain waves presented a pattern that was consistent with the HERA model predictions for healthy individuals. The results showed prominent theta and delta responses over the frontal region, which were responsible for the long-lasting sensory stimuli representation. The results of the retrieval phase showed that the theta and delta bands were prominent at the right side of the frontal region, possibly responsible for matching the input visual with the sensory representations in the learning phase. The fact that the parietal and occipital EEG oscillations were not consistent with the HERA model showed the importance of incorporating multimodal neuroimaging modalities wherein different modalities can indicate different aspects of long-term memory retrieval.

There is no standard test used for the detection of handedness. This could be considered a limitation of the study and will be considered in future work.

## Figures and Tables

**Table 1:** The 36 electrodes (16 pairs) used in the experiment (augmented a 10–20 system).

Left Brain Lobes	Right Brain Lobes
F1 (19)	F2 (4)
F3 (24)	F4 (124)
F5 (27)	F6 (123)
F7 (33)	F8 (122)
F9 (32)	F10 (1)
FC1 (13)	FC2 (112)
FC3 (29)	FC4 (111)
FC5 (28)	FC6 (117)
FP1 (22)	FP2 (9)
FT7 (34)	FT8 (116)
FT9 (38)	FT10 (121)
O1 (70)	O2 (83)
P1 (60)	P2 (85)
P3 (52)	P4 (92)
P5 (51)	P6 (97)
P7 (58)	P8 (96)
PO3 (67)	PO4 (77)
PO7 (65)	PO8 (90)

**Table 2:** Mean ± SD values for HERA model for an alpha, beta, theta, delta, and gamma-band for the frontal, parietal, and occipital regions.

Brain Region					EEG Bands					
	Alpha		Beta		Theta		Delta		Gamma	
	HERA mean	HERA ±SD	HERA mean	HERA ±SD	HERA mean	HERA ±SD	HERA mean	HERA ±SD	HERA mean	HERA ±SD
Parietal Region	−6.92	±19.83	−21.83	±64.26	−9.15	±14.52	−2.38	±10.73	−6.23	±80.21
Frontal Region	−4.97	±17.57	−20.21	±50.32	13.44	±38.33	84.52	±54.63	−36.61	± 62.80
Occipital Region	13.99	±136.64	57.13	±469.12	8.46	±83.67	3.93	±54.97	84.89	±481.83

**Table 3:** *t*-test table for the frontal region (all bands) of the HERA model.

	Tstat	Df	Sd	*p*	Effect Size
**theta**	**3.04**	**30**	**24.99**	**0.004**	**0.55**
**delta**	**13.30**	**30**	**35.95**	**0.0001**	**2.34**
**alpha**	1.03	30	26.71	0.307	0.18
**beta**	0.86	30	129.68	0.392	0.16
**gamma**	1.06	30	194.72	0.295	0.20

**Table 4:** *t*-test table for the parietal region (all bands) of the HERA model.

	Tstat	Df	Sd	*p*	Effect Size
**beta**	**2.10**	**30**	**86.74**	**0.043**	**0.34**
**delta**	2.38	30	21.33	0.023	0.43
**alpha**	1.66	30	26.72	0.106	0.30
**theta**	1.02	30	13.16	0.314	0.19
**gamma**	0.92	30	137.52	0.361	0.17

**Table 5:** *t*-test table for the occipital region (all bands) of the HERA model.

	Tstat	Df	Sd	*p*	Effect Size
**theta**	**0.57**	**30**	**83.67**	**0.571**	**0.10**
**delta**	0.40	30	54.97	0.688	0.08
**alpha**	0.57	30	136.64	0.566	0.10
**beta**	0.68	30	469.12	0.496	0.12
**gamma**	0.99	30	481.83	0.326	0.18
